# Tunable Crystalline Phases in UV-Curable PEG-Grafted Ladder-Structured Silsesquioxane/Polyimide Composites

**DOI:** 10.3390/ma13102295

**Published:** 2020-05-15

**Authors:** Ryung Il Kim, Ju Ho Shin, Jong Suk Lee, Jung-Hyun Lee, Albert S. Lee, Seung Sang Hwang

**Affiliations:** 1 Materials Architecturing Research Center, Korea Institute of Science and Technology, Hwarangno 14–gil 5, Seongbuk Gu, Seoul 02792, Korea; kri0521@kist.re.kr; 2 Department of Chemical and Biological Engineering, Korea University, 5–1 Anam-dong, Seongbuk Gu Seoul 02850, Korea; leejhyyy@korea.ac.kr; 3 Department of Chemical and Biomolecular Engineering, Sogang University, Baekbeom-ro 35, Mapo-gu, Seoul 04107, Korea; jaess0@naver.com (J.H.S.); jsleesogang@gmail.com (J.S.L.)

**Keywords:** polyimide, ladder-structured polysilsesquioxane, hybrid composite, 6FDA-DAM:DABA (3:2)

## Abstract

A series of UV-curable hybrid composite blends containing a carboxylic acid functionalized polyimidewith varying amounts of high molecular weight (~1 K) PEG-grafted ladder-structured polysilsesquioxanes copolymerized with methacryl groups were fabricated and their structural, thermal, mechanical, and surface properties characterized. At a composite weight ratio of polyimide above 50 wt.%, a stark shift from amorphous to crystalline polyethylene glycol (PEG) phases were observed, accompanied by a drastic increase in both surface moduli and brittleness index. Moreover, fabricated composites were shown to have a wide range water contact angle, 9.8°–73.8°, attesting to the tunable surface properties of these amphiphilic hybrid polymer composites. The enhanced mechanical properties, combined with the utility of tunable surface hydrophilicity allows for the possible use of these hybrid polymer composites to be utilized as photosensitive polyimide negative photoresists for a myriad of semiconductor patterning processes.

## 1. Introduction

High performance polymers such as polyimides have been widely been researched for a myriad of applications requiring high thermal stability, low dielectric constant, high free fractional volume, low coefficient of thermal expansion, and high optical transparency [1,2,3]. Most notable application fields include photoresists [4], gas separation membranes [5], high temperature polymer composites [6], flexible display substrates [7], protective optical films [8], and microelectronics [9,10]. While exhibiting excellent properties by itself, in certain applications such as those in various semiconductor processes of fabricating patterned surfaces requiring photosensitive resist layers, polyimides and their organically modified variants have been reported to have some limitations in thermal, thermomechanical, and electrical properties [11]. Strategies to enhance such properties have often resorted to the hybridization method with inorganic or organic–inorganic hybrid materials [12,13]. The wide utility of polyimide-based hybrid composites is due to its tunable thermal, mechanical, optical properties through careful selection of polyimide and hybridization material.

Some examples of hybridization of polyimides include the conventional physical blending with metal oxide or inorganic materials. Joly reported a polyimide silica composite membrane by either blending silica to a polyamic acid polymer solution or by including a silica precursor, tetramethylorthosilicate (TMOS) followed by thermal imidization after casting as films [14]. Chen and coauthors [15] also expanded upon this material concept by increasing the silica content, albeit only up 40 wt.%. However, in both studies a high degree of interface heterogeneity between polyimide and silica limited the extent to which the hybridization strategy aimed to target the enhancement factor of selective gas permeation. Another widely reported method includes the use of aminosilane precursors to co-polymerize and imidize with anhydrides, leaving an alkoxysilane terminus to which sol-gel based hydrolysis–condensation networking of Si–O–Si bonds was possible. The Mark group [16] as well as the Iroh group [17] reported these in-situ sol-gel methods to improve upon the interfacial voids. However, even in such examples, the fractional inorganic component can only be used according its dispersibility and compatibility with polyimide matrix, thereby limiting its ability to enhance the target physical properties.

In order to improve upon the compatibility issues with inorganic materials, inorganic–organic hybrid materials such as silsesquioxanes have been touted as appropriate material due to both organic functional group and inorganic siloxane networks providing a bridge between both phases enabling high compatibility between organic polymer networks. While cage structured polyhedral oligomeric silsesquioxanes, commonly known as polyhedral oligomeric silsesquioxane (POSS) [18,19,20], have been widely reported as polyimide hybridization material [21], their low molecular weight, as well as relatively low solubility in organic solvents have limited their effectiveness as filler in many cases [22].

A new inorganic–organic hybrid material, ladder-structured polysilsesquioxanes [23,24], has been the subject of extensive works in our group, ranging from hybrid gel polymer electrolytes for lithium ion batteries [25,26,27,28,29], gas separation membranes [30,31], as well as low dielectric constant interlayer films [32]. The wide-ranging applications of these materials is due to their unique properties enabled by controlled Si–O–Si siloxane structure. The fully condensed nature of the ladder-like structure with high molecular weight endows polymeric properties controlled through organic functional group–R type and composition of different–R groups [33,34,35].

In designing a homogeneously compatible polyimide and ladder-like structured polysilsesquioxane hybrid composite, several factors have to be considered. As both polyimide and ladder-like polysilsesquioxane have different solubility parameters, polyimide and ladder-like polysilsesquioxanes were found to be immiscible unless a finely tuned interface between organic functional groups was designed [36]. In our previous paper, we noted how hydrogen bonding interactions between the carboxylic acid groups of hexfluoroisopropylidine diphthalic anhydride-co-diaminomesitylene-co-diaminobenzoic acid derived polyimide, 6FDA-DAM:DABA (3:2) (PI) and the pyridine groups of ladder-like polysilsesquioxanes enabled a high degree of compatibility over those pairs without any interactive forces [36,37]. As such, in the present study, we designed a high molecular weight polyethylene glycol, PEG (1 K) grafted ladder-like polysilsesquioxane that can enable hydrogen bonding interactions with the carboxylic acid groups of 6FDA-DAM:DABA (3:2) (PI). Copolymerization of methacryl groups allowed for UV-curing and an examination of their surface mechanical properties as a function of PEG crystalline phases controlled through a high ladder-like polysilsesquioxane composite fraction.

## 2. Experimental

### 2.1. Materials

Polyethylene glycol monomethyl ether 1000 (MPEG 1000) (Aldrich, *M_n_* = 1000) and potassium carbonate (K_2_CO_3_) (Aldrich, 99%) were dried at 40 °C under vacuum overnight before use. Tetrahydrofuran (THF) (Daejung, 99.9%) and dimethylformamide (DMF) (Daejung, 99.9%) were used after purification through a molecular sieve distillation device. Hexane (Aldrich, 99.9%) sodium hydride (NaH) (Aldrich, 60% dispersion in mineral oil), allyl bromide (Aldrich 98%), 2,2-dimethoxy-2-phenylacetophenone (DMPA) (Aldrich 99%), anhydrous magnesium sulfate (MgSO_4_) (Daejung), methylene chloride (M.C., 99.9%) (Daejung), polyethylene glycol dimethacrylate (PEGDMA) (Aldrich, Mn = 750), photoinitiator ethyl(2,4,6-Trimethylbenzoyl)-phenyl phosphinate (Omnirad TPO-L) (IGM) and polyimide, 6FDA-DAM:DABA (3:2) (Huntsman Chemicals) having molecular weight (*Mn* = 91 k, *Mw* = 18 K) were used as received. 3-mercaptopropyltrimethoxysilane (MPTMS) (Gelest) and 3-methacryloxypropyltrimethoxysilane (MMATMS) (Gelest) were distilled over CaH_2_ prior to use.

### 2.2. Synthesis of Allyl-Terminated PEG (MPEG-Allyl)

In a 250 mL 3-neck round-bottom flask, MPEG 1000 (10 g, 0.01 mol) and THF (100 mL) were charged and stirred for 30 min. And then NaH (0.026 g, 0.011 mol) was added. When the color of the solution was yellow, allyl bromide (3.63 g, 0.03 mol) was added. After purging with N_2_, the reaction was stirred for 12 h at a temperature of 40 °C. Afterwards, the solid contents of were settled while centrifuging at 4000 rpm for about 30 min. After that the liquid material is separately poured and collected into 100 mL vial. THF was removed through a rotary evaporator. The crude product was dissolved in MC (30 mL) and extracted with deionized water (90 mL) several times. And then only MC solution was collected using a separate funnel. The collected MC solution was dropped in a hexane (1.5 L) with ice bath. After filtering with a Buchner funnel and evaporation of dichloromethane in a vacuum oven at room temperature for 12 h, a white powder (9 g, 90% crude yield) was obtained.

^1^H NMR (CDCl_3_, ppm):

3.40–3.42 (s, CH_2_CHCH_2_O(CH_2_CH_2_O)_22_**CH_3_**, 3 H),

3.6–3.7 (m, CH_2_CHCH_2_O(**CH_2_CH_2_**O)_22_CH_3_, 88 H),

4.03–4.08 (m, CH_2_CH**CH_2_**O(CH_2_CH_2_O)_22_CH_3_, 2 H),

5.18–5.34 (m, **CH_2_**CHCH_2_O(CH_2_CH_2_O)_22_CH_3_, 2 H),

5.89–6.0 (m, CH_2_**CH**CH_2_O(CH_2_CH_2_O)_22_CH_3_, 1 H).

### 2.3. Synthesis of Trimethoxysilane-Terminated PEG (MPEGTMS)

In a 100 mL round-bottom flask, MPEG-Allyl (10 g, 0.01 mol) and THF (50 mL) were charged and stirred to completely dissolve. Then, MPTMS (5.89 g, 0.03 mol) and DMPA (0.05 g, 5 wt.%) were added and dissolved completely. After confirming that it is completely dissolved, the flask was purged by N_2_. After placing it in a UV black box, the reactant solution was exposed by 6 UV-lamps (λ = 254 nm) to react for 3 h. And then the solution is dropped dropwise into hexane (1.5 L) chilled under an ice bath. The powder was filtered and dried in a vacuum oven at room temperature to obtain a white powder (9.9 g, 99%).

^1^H NMR (CDCl_3_, ppm):

0.72–0.79 (m, CH_3_)_3_Si**CH_2_**CH_2_CH_2_SCH_2_CH_2_CH_2_O(CH_2_CH_2_O)_22_CH_3_, 2 H),

1.64–1.74 (m, (CH_3_)_3_SiCH_2_**CH_2_**CH_2_SCH_2_CH_2_CH_2_O(CH_2_CH_2_O)_22_CH_3_, 2 H),

1.75–1.95 (m, (CH_3_)_3_SiCH_2_CH_2_CH_2_SCH_2_**CH_2_**CH_2_O(CH_2_CH_2_O)_22_CH_3_, 2 H),

2.47–2.64 (m, (CH_3_)_3_SiCH_2_CH_2_**CH_2_**S**CH_2_**CH_2_CH_2_O(CH_2_CH_2_O)_22_CH_3_, 4 H),

3.37–3.39 (s, (CH_3_)_3_SiCH_2_CH_2_CH_2_SCH_2_CH_2_CH_2_O(CH_2_CH_2_O)_22_**CH_3_**, 3 H),

3.53–3.55 (m, (CH_3_)_3_SiCH_2_CH_2_CH_2_SCH_2_CH_2_**CH_2_**O(CH_2_CH_2_O)_22_CH_3_, 2 H),

3.56–3.58 (m, (**CH_3_**)_3_SiCH_2_CH_2_CH_2_SCH_2_CH_2_CH_2_O(CH_2_CH_2_O)_22_CH_3_, 9 H),

3.6–3.7 (m, (CH_3_)_3_SiCH_2_CH_2_CH_2_SCH_2_CH_2_CH_2_O(**CH_2_CH_2_**O)_22_CH_3_, 88 H)

### 2.4. Synthesis of LPEOMASQ82

Synthesis of ladder-structured (polyethylene glycol-co-methylacryloxypropyl) silsesquioxane LPEGMASQ82, with polyethylene glycol: methacryl ratio 8:2, was conducted following a known literature procedure [24,38]. Typically, in a 100 mL round bottom flask, potassium carbonate, K_2_CO_3_ (14 mg, 0.1 mmol) was dissolved in H_2_O (1.68 g, 93.2 mmol) and DMF (10 g) added. The solution was stirred at room temperature until transparent. To this solution, previously synthesized trimethoxysilane-terminated PEG (MPEGTMS) (26.77 g, 22.4 mmol) and 3-methacryloxypropyltrimethoxysilane (MMATMS) (1.39 g, 5.6 mmol) was added under nitrogen. The reaction mixture was stirred vigorously for five days or until the molecular weight reached its maximum value under a water bath of 40 °C. After evaporation of DMF, the light brown tacky solid was dissolved in dichloromethane and extracted with water at least twice to remove the base catalyst. After adding anhydrous magnesium sulfate to remove residual water in the organic layers, filtering, and evaporation of MC, the light brown solid LPEGMASQ82 was obtained (22 g, 88% yield).

^1^H NMR (CDCl_3_, ppm):

0.55–0.95 (m, Si**CH_2_**CH_2_CH_2_OCOCCH_2_CH_3_,

Si**CH_2_**CH_2_CH_2_SCH_2_CH_2_CH_2_O(CH_2_CH_2_O)_22_CH_3_, 4 H),

1.2–1.4 (m, SiCH_2_**CH_2_**CH_2_OCOCCH_2_CH_3_, 2 H),

1.58–1.92 (m, SiCH_2_**CH_2_**CH_2_SCH_2_**CH_2_**CH_2_O(CH_2_CH_2_O)_22_CH_3_, 4 H),

1.93–2.0 (m, SiCH_2_CH_2_CH_2_OCOCCH_2_**CH_3_**, 3 H),

2.45–2.7 (m, SiCH_2_CH_2_**CH_2_**S**CH_2_**CH_2_CH_2_O(CH_2_CH_2_O)_22_CH_3_, 4 H),

3.37–3.39 (m, SiCH_2_CH**_2_**CH_2_SCH_2_CH_2_CH_2_O(CH_2_CH_2_O)_22_**CH_3_**, 3 H),

3.53–3.55 (m, SiCH_2_CH_2_CH_2_SCH_2_CH_2_**CH_2_** O(CH_2_CH_2_O)_22_CH_3_, 2 H),

3.6–3.7 (m, SiCH_2_CH_2_CH_2_SCH_2_CH_2_CH_2_O(**CH_2_CH_2_**O)_22_CH_3_, 88 H),

4.05–4.20 (m, SiCH_2_CH_2_**CH_2_**OCOCCH_2_CH_3_, 2 H),

5.5–6.2 (m, SiCH_2_CH_2_CH_2_OCOC**CH_2_**CH_3_, 2 H).

^29^Si NMR (CDCl_3,_ ppm): -55 to -66 ppm, -66 to -80 ppm.

### 2.5. Fabrication of Composites

To make polymer solutions, LPEGMASQ82 and 6FDA-DAM:DABA (3:2) (PI) were added to dry glass vials at a weight ratio of 10:0, 1:9, 3:7, 5:5, 7:3, 9:1, and 0:10 respectively. After that, tetrahydrofuran (THF) was added to prepare a 10 solid wt.% concentration. To this solution, PEGDMA was added in corresponding 2 equivalents of the methacryloxypropyl groups of LPEGMASQ82 along with 1 wt.% contents of photoinitiator Omnirad TPO-L. These solutions were mixed using a vortex and sonicated for 1 h. Afterwards, these solutions were cast with a 50 um doctor blade on slide glasses and silicon wafers. Fabricated composites were left at room temperature overnight and dried in a vacuum oven at 40 °C for 2 h. Finally, the coated composites were irradiated with a HITACHI UV-lamp system with a lamp intensity of 100 mW/cm^2^, with a total UV energy output of 3 J/cm^2^.

### 2.6. Characterization

Number average molecular weight (*M_n_*) and molecular weight distributions (*M_w_*/*M_n_*) of the polymers were measured by JASCO PU-2080 plus gel permeation chromatography (GPC) system equipped with refractive index detector (RI-2031 plus), UV detector (λ = 254 nm, UV-2075 plus), and Viscotek SLS apparatus using THF as the mobile phase at 40 °C with a flow rate of 1 mL/min. The samples were separated through four columns (Shodex-GPC KF-802, KF-803, KF-804, KF-805). NMR spectra were recorded in CDCl_3_ at 25 °C on a Bruker Avance (^1^H: 400 MHz, ^29^Si: 79.5 MHz). FTIR spectra were measured using Perkin–Elmer FTIR system (Spectrum-GX) using solvent cast films on polished KBr plates. Thermal gravimetric analysis (TGA) was performed by TA Instrument TGA 2950 under N_2_ at a temperature ramp rate of 10 °C/min. Differential scanning calorimetry was carried out with a TA Instrument DSC Q20-1426 using a heating rate of 10 °C / min under N_2_ atmosphere. Note that second scans were only shown. Nanoindentation measurements were conducted on a Hysitron Inc. TriboIndenter equipped with a Berkovich diamond tip [34,39]. Measurements of elastic modulus were performed as a continuous stiffness measurement on samples coated on silicon wafer at thickness of 5 μm. Optical microscopic images were obtained using a polarization microscope Lecica DM2500P. UV curing experiments were carried out on a Hitachi UV-Spot Cure system at UV irradiation wavelength of 365 nm. UV transmittance values were measured with a JASCO V-730 instrument. The d-spacing values of dense sample films was measured by wide angle x-ray diffraction (WAXD, Dmax2500/PC (Rigaku)) with Cu Kα radiation (λ = 1.5406 Å). The surface contact angle was measured by contact angle analyzer.

## 3. Results and Discussion

### 3.1. Synthesis of LPEGMASQ82 and Fabrication of Hybrid Composites

Unlike ladder structured polysilsesquioxanes grafted with a PEG molecular weight of ~300 described in our previous study [40], ladder-structured polysilsesquioxanes with a long chain PEG (molecular weight of ~1 K) was synthesized to see the effect of PEG crystallinity within a hybrid composite film with polyimide. Here, the PEG functions as compatibilizing functional group enabling multiple hydrogen bonding interactions between the ethylene oxide chain and carboxylic acid of the 6FDA-DAM:DABA (3:2) polyimide matrix.

**Scheme 1:**
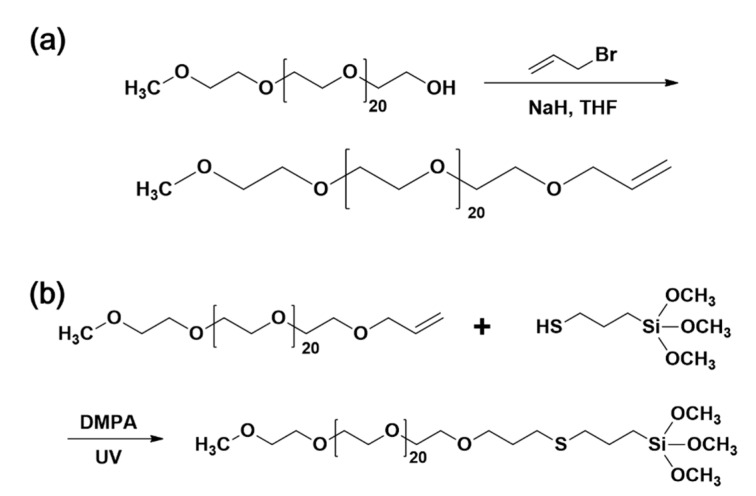
Synthesis of (**a**) allyl terminated PEG and (**b**) Trimethoxysilane-terminated PEG.

**Figure 1:**
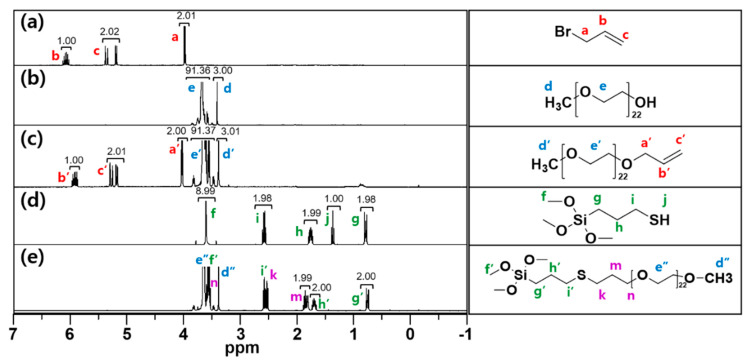
^1^H NMR spectra of (**a**) allyl bromide, (**b**) PEG, (**c**) polyethylene glycol monomethyl ether (MPEG), (**d**) 3-mercaptopropyltrimethoxysilane (MPTMS) and (**e**) MPEGTMS and corresponding chemical structure assignments.

For this, we first synthesized an allyl terminated PEG, through SN_2_ addition reaction of monohydroxy terminated PEG with allyl bromide, Scheme 1a followed by thiol-ene click reaction with 3-mercaptopropyltrimethoxysilane Scheme 1b to yield a trimethoxysilane-terminated PEG. Figure 1 shows the ^1^H NMR spectra of trimethoxysilane-terminated PEG, MPEGTMSreagents and products. The proton peak of (a’), (b’) and (c’) from 4 to 6 ppm were assigned to the allyl group of MPEG-Allyl Figure 1c. These results indicated that MPEG was completely allylated to become MPEG-Allyl as described Scheme 1a. In Figure 1e, proton peaks (e”) at 3.6 ppm and (d”) at 3.55 ppm were represented from MPEG of MPEGTMS and proton peak (j) at 1.35 ppm from thiol of MPTMS Figure 1d disappeared. These results suggested that MPTMS was fully reacted to PEGTMS by thiol-ene reaction as described Scheme 1b.

**Scheme 2:**
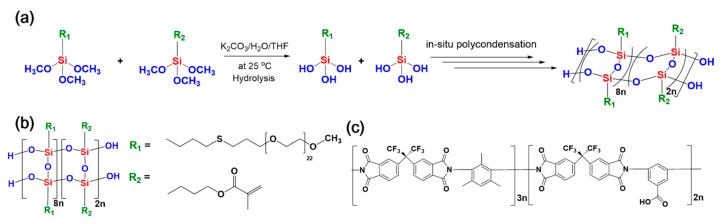
(**a**) Synthesis of LPEGMASQ82 and chemical structures of (**b**) LPEGMASQ82, and (**c**) 6FDA-DAM:DABA (3:2) (PI).

**Figure 2:**
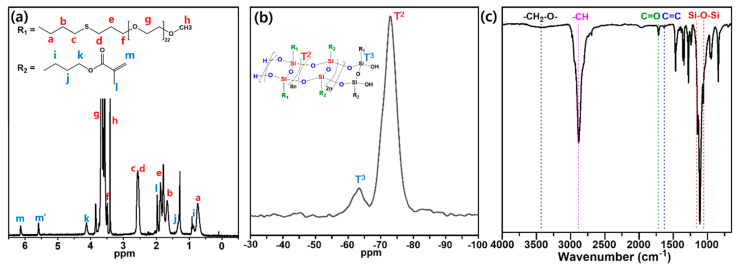
(**a**) ^1^H NMR, (**b**) ^29^Si NMR, and (**c**) FTIR spectrum of LPEGMASQ82.

Using MPEGTMS, we then copolymerized it with 3-methacryloxypropyltrimethoxysilane (MMATMS) at a monomer initial feed ratio of 8:2 to yield LPEGMASQ82 based on our previously described synthesis method Scheme 2a [40]. MMATMS was introduced to the synthesis of LPEGMASQ82 for UV-curing. The prepared LPEGMASQ82 was characterized using GPC, ^1^H NMR, ^29^Si NMR, and FTIR techniques as shown Table 1 and Figure 2. As shown Table 1, it was confirmed that LPEOMA82 had molecular weights (*M_w_*) of 12,300, thus a high molecular weight characteristic of ladder structured polysilsequioxanes [24]. In the ^1^H NMR spectra, the peaks (m) and (m’) from 5 to 6 ppm were assigned to the C=C bond of methacryl moiety and the peak (g) at 3.5 ppm represents ethylene oxide protons of PEG Figure 2a.

Furthermore, ^29^Si NMR analysis reveals the structure of the siloxane backbone of LPEGMASQ82. The T^2^ (−63 ppm) peak of the backbone terminal portion (R-Si(OSi-)_2_OH) of the LPEGMASQ82 and the T^3^ (−72 ppm) peak of the main backbone portion (R-Si(OSi-)_3_) of the LPEGMASQ82 were confirmed as seen in Figure 2b. This result shows the characteristic Si peaks of ladder structured polysilsequioxane [24,40] were fully condensed. Moreover, as shown Figure 2a, proton peaks of Si-OH groups at 5.0 ppm were absent. These analyses indicate that all methoxy groups of PEGTMS and MMATMS were fully hydrolyzed, condensed and formed to LPEGMASQ82 with ladder structure siloxane bond. FTIR analysis was also conducted as shown in Figure 2c. LPEGMASQ82 exhibits two strong absorption peaks, which indicate ladder structure, located at 1150 cm^−1^ and 1050 cm^−1^ assigned to the asymmetrical horizontal (–Si–O–Si–) and vertical (–Si–O–Si–) siloxane bonds [23]. Also the C=O carbonyl and C=C bond of methacyloxypropyl groups at 1715 cm^−1^ and 1635 cm^−1^ were assigned to C–H stretching absorption peak at 2880 cm^−1^ from PEG. These results indicate that LPEGMASQ82 was successfully synthesized. GPC analysis of mPEG, allyl-mPEG, mPEG-TMS, as well as LPEGMASQ82 are shown in Supplementary Materials Figure S1 with LPEGMASQ82 giving high molecular weight and monomodal peak indicative of ladder structure and not mixture of cage silsesquioxanes.

**Figure 3:**
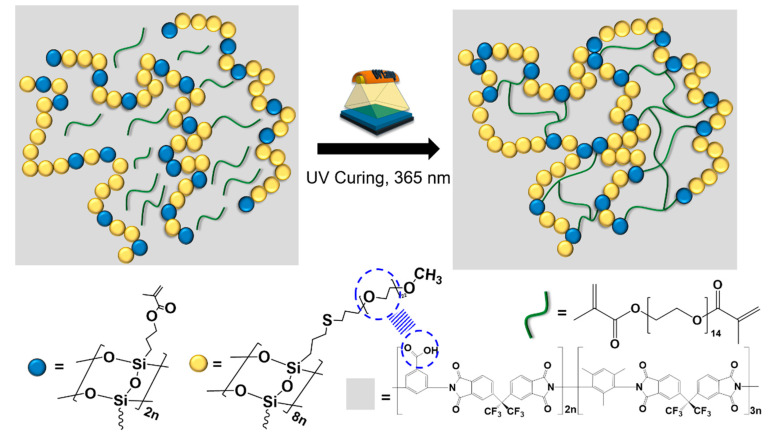
Schematic of UV curing process for hybrid composite and interactions between PI and LPEGMASQ82.

The fabricated composites were blended with different ratios 6FDA-DAM:DABA (3:2) (PI), LPEGMASQ82 and PEGDMA as shown Table 2. After making polymer solutions in THF, they were cast onto glass and silicon wafer substrates with a 50 um preset doctor blade and cured by UV lamp (365 nm) with 1 wt.% contents of photoinitiator Omnirad TPO-L Figure 3. The result of UV curing was monitored by FTIR. Figure S2 in the Supplementary Materials exhibits the characteristic peak of FTIR spectrum for before and after the UV curing of the PI and LPEGMASQ82 composite. As shown, C=C peak at 1635 cm^−1^ derived from the methacryl moiety was disappeared completely after UV curing and a high degree of densification and increase in hardness was observed without decrease in optical transparency. Such properties will be discussed in detail in later sections. Moreover, the specific hydrogen bonding interactions between the carboxylic acid of PI and the ethylene oxide repeating units of LPEGMASQ82 as depicted in Figure 3 enabled a high degree of compatibility between PI and LPEGMASQ82, as shown by the FTIR peak shift assigned to the C O vibration band shifting to 1723 cm^−1^_,_ which was between that for PI at 1731 cm^−1^ and LPEGMASQ82 at 1715 cm^−1^ as shown by Figure S3 in the Supplementary Materials.

### 3.2. Thermal and Structural Characterization

**Figure 4:**
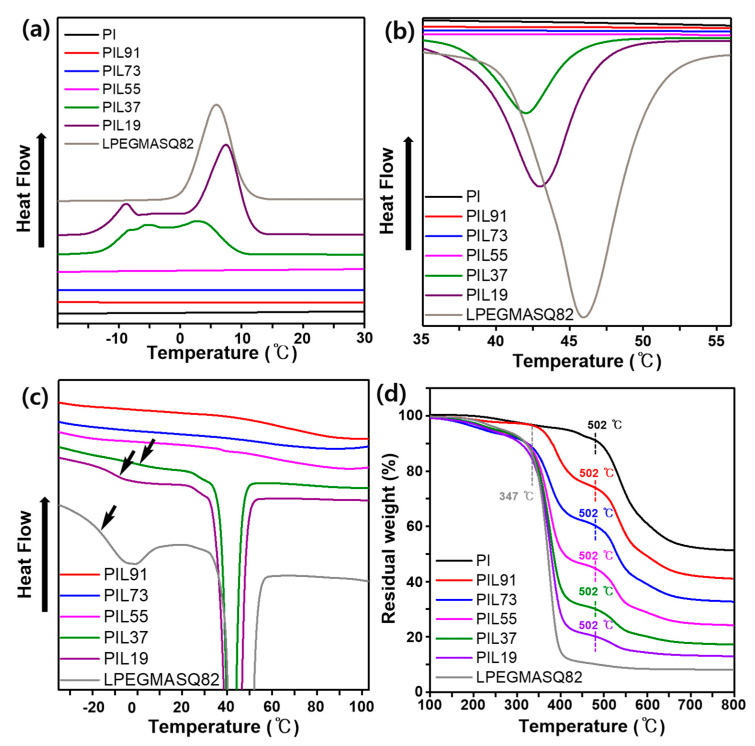
DSC thermograms of hybrid composites showing expanded views of: (**a**) T_c_, (**b**) T_m_, and (**c**) T_g_, and thermogravimetric analysis (TGA) of hybrid composites (**d**).

The thermal properties of the hybrid composites were evaluated by DSC and TGA. As shown in Figure 4a,b, neat LPEGMASQ82 had sharp crystallization temperature (T_c_) and melting temperature (T_m_) which were attributed to the crystalline PEG phases at 11 and 46 °C, respectively. This is noteworthy as these thermal phase transitions were not seen for ladder-structured polysilsesquioxanes grafted with low molecular weight PEG as in our previous study [40]. For the hybrid composites at low LPEGMASQ82 weight fractions lower than 5:5, we did not observe characteristics PEG melting or crystallization transitions, as the amorphous polyimide matrix sufficiently suppressed the crystallinity of PEG phases. However, for blended hybrid composite samples with LPEGMASQ82 portion 7:3 and higher, we clearly observed the melting and crystallization thermal phase transitions. This interesting thermal behavior was further reflected in the disappearance of thermal glass transition temperature (T_g_) for those same hybrid composite samples without distinct crystallization and melting transitions, indicating a higher degree of miscibility and compatibility between polyimide matrix Figure 4c and high molecular weight PEG grafted ladder-structured polysilsesquioxane. TGA analysis was also used to characterize the thermal degradation behavior. As shown in Figure 4d, all materials exhibited exceptionally high thermal stability. As both ladder-structured polysilsesquioxane and polyimide materials are known to be thermally stable thermoplastic materials and high degree of compatibility [24], the possibility for use in high temperature processing applications can be considered another advantage of the developed hybrid composites.

**Figure 5:**
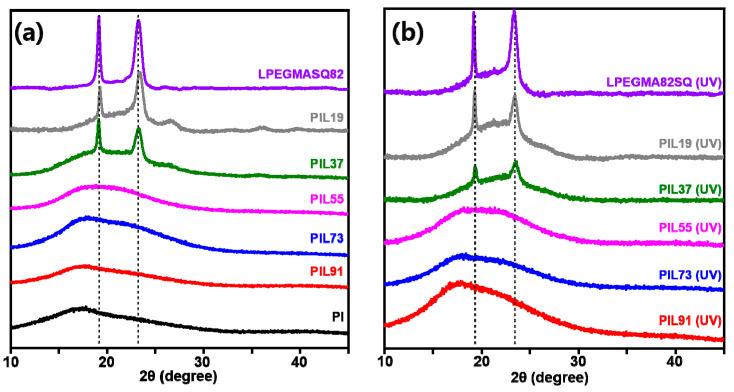
XRD spectra of hybrid composites: (**a**) before UV-curing and (**b**) after UV-curing.

To characterize the structure of the hybrid composite films further, XRD analysis was carried out as shown in Figure 5. As shown, LPEGMASQ82 showed a broad peak from 16° to 25°, representing an amorphous siloxane backbone [24], and two additional sharp peaks from the grafted PEG at 19° and 23° regardless of UV curing [41]. These results indicated that LPEGMASQ82 had crystallinity due to grafted high molecular weight PEG (~1 K). When blending from PIL91 to PIL55, the crystalline XRD peaks derived from the PEG moieties of LPEGMASQ82 were not shown, reflecting the suppression of PEG crystallinity via the polyimide matrix.

### 3.3. Optical Properties of Hybrid Composites

**Figure 6:**
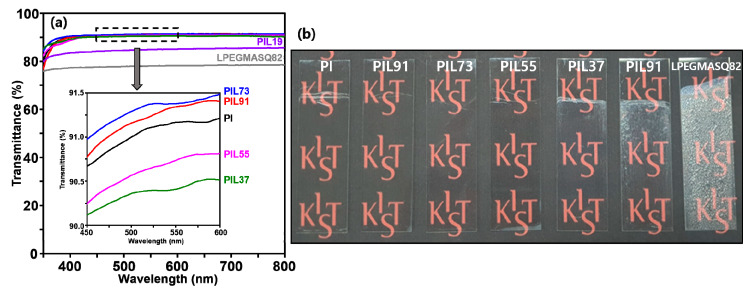
(**a**) UV-vis spectra of hybrid composites and (**b**) digital photographs of hybrid composites coated on glass slides.

The optical properties of 6FDA-DAM:DABA (3:2) (PI)**,** LPEGMASQ82, and fabricated hybrid composites were analyzed via UV-vis spectroscopy and polarized light microscopy. As shown in Figure 6a, the fabricated hybrid composites as well as neat PI exhibited a high degree of optical transparency (>90% at 550 nm). By comparison, LPEGMASQ82 exhibited an optical transmittance of only 78%. When visually inspecting the glass slide coated samples Figure 6b, we clearly observed the increased optical birefringence of LPEGMASQ82 compared to the other samples, which we attributed to the crystalline phases observed in the previous XRD results. By slowly adding PI into LPEGMASQ82, the optical transparency increased to 80% for PIL19 and exceeded 90% from PIL37. It was also noteworthy to point out that the optical transmittance even surpassed pure PI when PI content was less than 5:5. This phenomenon was attributed not only to the polyimide matrix suppressing PEG crystalline phases, but also the Si–O–Si siloxane network functioning to amorphize PEG and total composite as a whole to reduce birefringence of stemming from the π–π stacking interactions of PI.

**Figure 7:**
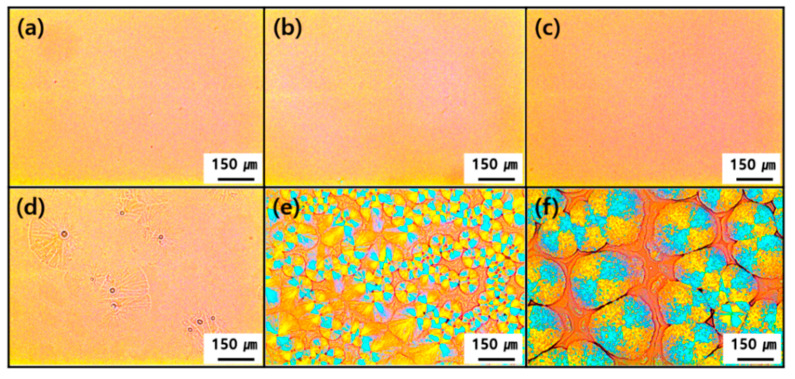
Polarized light microscopy images of (**a**) PIL91, (**b**) PIL73, (**c**) PIL55, (**d**) PIL37, (**e**) PIL19, and (**f**) LPEGMASQ82.

Polarized light microscopy images as shown in Figure 7 provide insight into the PEG crystalline domain sizes. Consistent with our previous spectroscopic evidence, no PEG crystalline phases were shown for hybrid composites with low LPEGMASQ82 content such as PIL91, PIL73, and PIL55. However, in PIL37, we observed small PEG spherulitic domains beginning to form. While these PEG spherulitic domains were not fully developed nor well-defined for PIL37, PIL19 exhibited a high degree of PEG crystalline domains inter-dispersed evenly throughout the PI matrix. As shown, the PEG spherulites’ average size was about 70 μm. Also, with neat LPEGMASQ82 we were able to see much larger crystal domains of about 200 μm. It was interesting to note that the segregated PEG crystalline domains from LPEGMASQ82 could be easily tuned according to blend ratio.

### 3.4. Surface Mechanical and Wetting Properties

**Figure 8:**
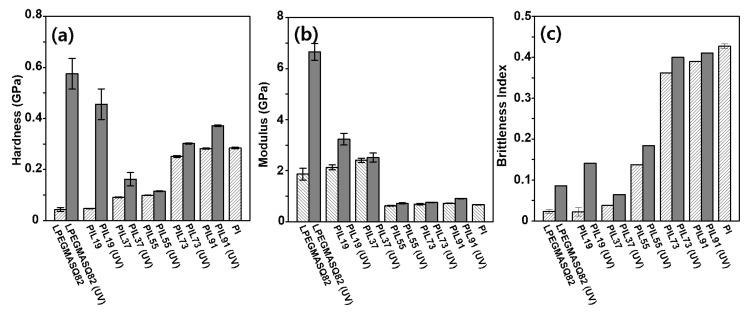
Nanoindentation results for hybrid composites showing measured: (**a**) hardness, (**b**) modulus, and (**c**) brittleness indices.

The surface mechanical properties of hybrid composites were examined by nanoindentation. As shown Figure 8, the hardness (*H*) and modulus (*E*) of hybrid composites coated silicon wafers were measured before and after UV curing and their brittleness index were calculated by dividing the hardness (*H*) by modulus (*E*) (brittleness index = *H/E*) [34,39]. The hardness values slightly decreased with the addition of the LPEGMASQ82 from neat PI to PIL73, but there was a significant decrease between PIL73 and PIL55. The decrease in hardness as the content of LPEGMASQ82 increases is due to the role of PEG of LPEGMASQ82 in the hybrid composites matrix as plasticizer. It is noteworthy to point out that the hardness value also varies significantly beginning at 50% LPEGSQMA82 content in hybrid composites, attributed to the stark shift from high compatibility to low compatibility, which was previously confirmed via thermal analysis and XRD. After UV crosslinking, the hardness values increased overall than before, and increased about 10 times in the case of pure LPEGMASQ82 (from 0.043 GPa up to 0.58 GPa) and PIL19 (from 0.047 to 0.456 GPa), which is related to the high crosslink density due to the high ratio of methacryl groups from LPEGMASQ82 and PEGDMA Figure 8a. Comparably, as LPEGMASQ82 content increased both before and after UV curing, the modulus values only slightly decreased from neat PI to PIL55 (from 0.665 to 0.625 GPa) and then gradually decreased from PIL37 to LPEGMASQ82 after the significant increase between PIL55 (0.625 GPa) and PIL37 (2.407 GPa) as shown in Figure 8b. This behavior was again attributed to the particular LPEGMASQ82 content to which phase separation occurs (more than 50%) and crystal domains of LPEGMASQ begin to form. Due to these crystalline domains of LPEGMASQ82, the modulus values drastically increased as the hybrid composites shifted from homogeneously compatible to having crystalline regions to which the surface brittleness increases [42]. This was clearly observed when analyzing the brittleness indices after UV-curing Figure 8c. These results are interesting in that we were able to show how mechanical properties of hardness, modulus and brittleness index were related to PEG chain as plasticizer and miscibility between PI and LPEGMASQ in the hybrid composites matrix.

**Figure 9:**
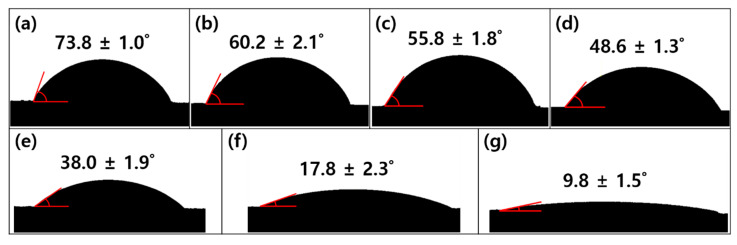
Water contact angles for: (**a**) PI, (**b**) PIL9:1, (**c**) PIL73, (d) PIL55, (**e**) PIL3:7, (**f**) PIL19, and (**g**) LPEGMASQ82**.**

The water contact angles of the hybrid composites were measured in order to investigate the wetting properties. As shown in Figure 9, the water contact angle decreased from PI to LPEGMASQ82 in order of 73.8°, 60.2°, 55.8°, 48.6°, 38.0°, 17.8°, and 9.8° with increasing LPEGMASQ82 content in the hybrid composites. These results indicated that the higher the content of LPEGMASQ82 with grafted PEG chains in the hybrid composites, the more hydrophilic it is. The hybrid composites showed a wide range of water contact angles according to the ratio of PI and LPEGMASQ82, which shows that the hydrophilicity and wettability can be controlled.

## 4. Conclusions

In conclusion, we were able to fabricate polyimide/ladder-like polysilsesquioxane hybrid composite films with tunable surface, mechanical, and optical properties according to the PEGylated ladder-like polysilsesquioxane weight fraction, which could be introduced at high loadings (up to 90 wt.%). The fabricated hybrid nanocomposites all exhibited high optical transparency, thermal stability, and at a PEGylated ladder-like polysilsesquioxane composition of 70%, the presence of PEG crystalline phases were observed. These crystalline phases greatly affected the surface mechanical and optical properties. The mechanical reinforcing abilities of the ladder-like polysilsesquioxane, as well as the tunable surface hydrophilicity allows for these hybrid nanocomposites to be utilized as photosensitive polyimide photoresist materials.

## Figures and Tables

**Table 1:** Molecular weight and molecular weight distributions for selected materials.

Sample	*M_n_* ^a^	*M_n_* ^b^	*M_w_* ^c^	PDI^d^
mPEG	1,037	1,036	1,100	1.06
Allyl-mPEG	1,077	1,071	1,100	1.06
mPEG-TMS	1,276	1,303	1,400	1.06
LPEGMASQ82	-	11,000	12,300	1.12

^a^ Number-average molecular weight (*M_n_*) calculated by ^1^H NMR, ^b^ Number-average molecular weight (*M_n_*) analyzed by GPC with polystyrene standard, ^c^ Weight-average molecular weight (*M_w_*) analyzed by GPC with polystyrene standard, ^d^ Polydispersity index (*M_w_*/*M_n_*).

**Table 2:** Blend ratio of hybrid composites.

Weight Ratio of Hybrid Composite Blend			
Sample Name	LPEGMASQ82	6FDA-DMA:DABA(3:2)	PEGDMA
LPEGMASQ82	100	0	2.5
PIL19	90	10	1.75
PIL37	70	30	1.5
PIL55	50	50	1.25
PIL73	30	70	0.75
PIL91	10	90	0.25
PI	0	100	

## Supplementary Materials

The following are available online at https://www.mdpi.com/1996-1944/13/10/2295/s1, Figure S1: Gel permeation chromatography (GPC) analysis in THF of mPEG, allyl-mPEG, mPEG-TMS, and LPEGMASQ82, Figure S2: FTIR spectra of hybrid composite before and after UV curing, Figure S3: Expanded FTIR spectra for LPEGMASQ82, PI, and Hybrid composite after UV-curing.
