# Supplementary material for: Tunable Crystalline Phases in UV-Curable PEG-Grafted Ladder-Structured Silsesquioxane/Polyimide Composites
Source: Materials (Basel). 2020 May 15;13(10):2295. doi: 10.3390/ma13102295 (PMC7287875; doi:10.3390/ma13102295)
Supplement: Supplementary file 1 [file materials-13-02295-s001.pdf]

materials

Supplementary Materials

Tunable Crystalline Phases in UV-Curable PEG-grafted Ladder-structured Silsesquioxane / Polyimide Composites

Ryung Il Kim [1,2], Ju Ho Shin [3], Jong Suk Lee [3], Jung-Hyun Lee [2], Albert S. Lee [1,*] and Seung Sang Hwang [1,*]

[1] Materials Architecturing Research Center, Korea Institute of Science and Technology, Hwarangno 14-gil 5, Seongbuk Gu, Seoul 02792, Republic of Korea; kri0521@kist.re.kr (R.I.K)
[2] Department of Chemical and Biological Engineering, Korea University, 5-1 Anam-dong, Seongbuk Gu 02850, Republic of Korea; leejhyyy@korea.ac.kr (J.H.L)
[3] Department of Chemical and Biomolecular Engineering, Sogang University, Baekbeom-ro 35, Mapo-gu, Seoul 04107, Republic of Korea; jaess0@naver.com (J.H.S); jsleesogang@gmail.com (J.S.L)
* Correspondence: aslee@kist.re.kr (A.S.L); sshwang@kist.re.kr (S.S.H) Tel.: +82-2-958-5335 (A.S.L); +82-2-958-5314 (S.S.H)

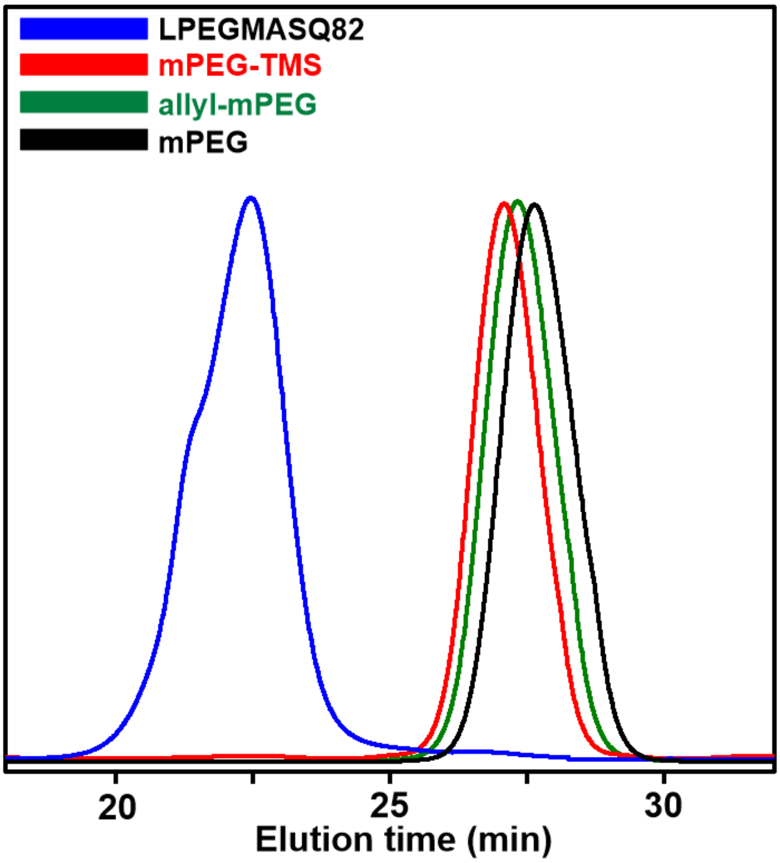

Figure S1. Gel permeation chromatography (GPC) analysis in THF of mPEG, allyl-mPEG, mPEG-TMS and LPEGMASQ82.

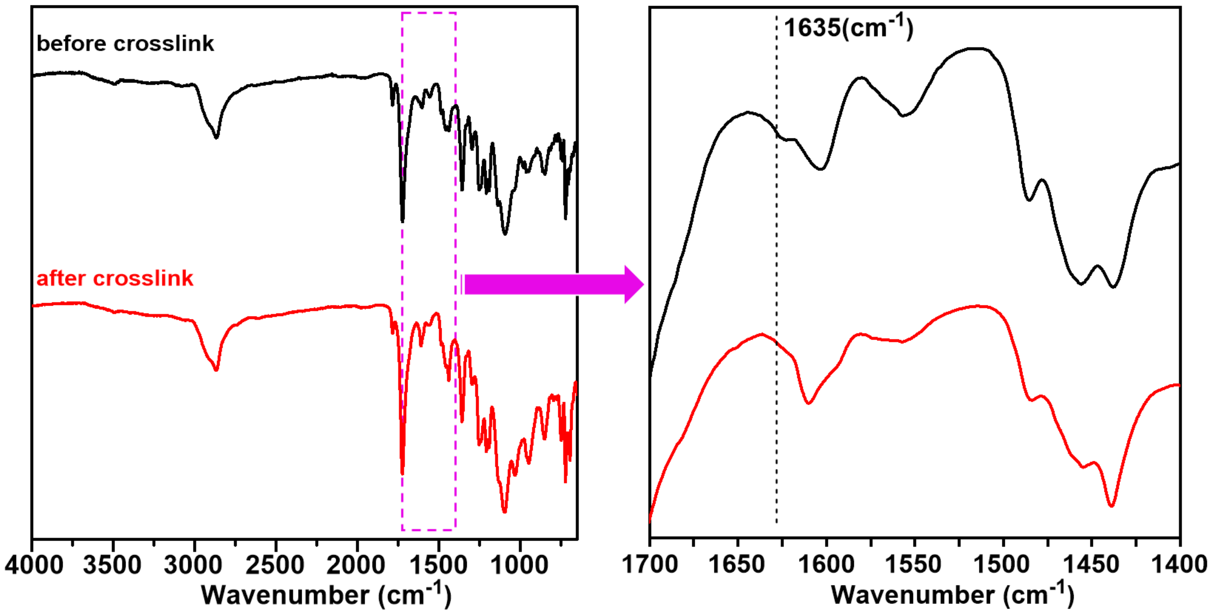

Figure S2. FTIR spectra of hybrid composite before and after UV curing.

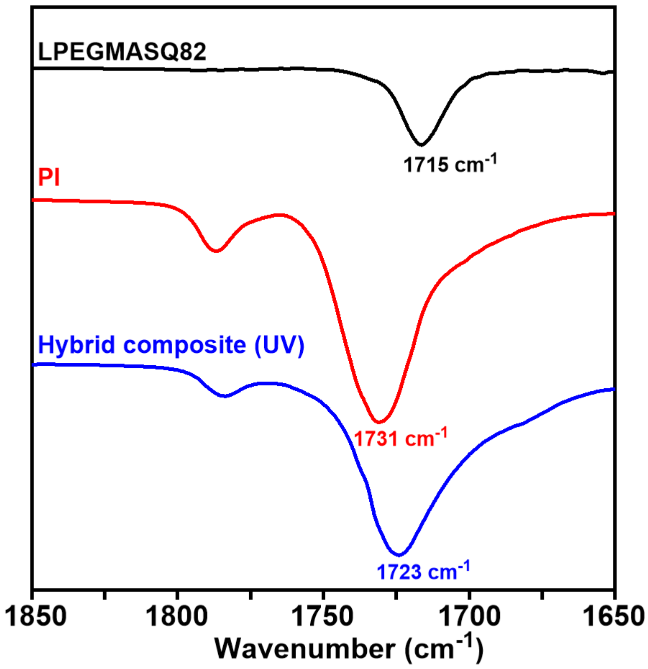

Figure S3. Expanded FTIR spectra for LPEGMASQ82, PI, and hybrid composite after UV-curing.

© 2020 by the authors. Submitted for possible open access publication under the terms and conditions of the Creative Commons Attribution (CC BY) license (http://creativecommons.org/licenses/by/4.0/).
